# Ecological adaptability and population growth tolerance characteristics of *Carex cinerascens* in response to water level changes in Poyang Lake, China

**DOI:** 10.1038/s41598-021-84282-x

**Published:** 2021-03-01

**Authors:** Xiaochen Yao, Yun Cao, Guodi Zheng, Adam T. Devlin, Xiao Li, Menghan Li, Siwen Tang, Lingming Xu

**Affiliations:** 1 School of Geography and Environment, Jiangxi Normal University, Nanchang, 330022 China; 2 Center for Environmental Remediation, Institute of Geographic Sciences and Natural Resources Research, Chinese Academy of Sciences, Beijing, 100101 China; 3 Key Laboratory of Poyang Lake Wetland and Watershed Research, Ministry of Education, Jiangxi Normal University, Nanchang, 330022 China

**Keywords:** Freshwater ecology, Grassland ecology, Wetlands ecology

## Abstract

Water level conditions are the key factors that affect the growth and distribution of wetland plants. Using *Carex cinerascens* (*C. cinerascens*) as the study species, we employ indoor simulations and field surveys. Our results show that *C. cinerascens* can adapt to rhythmic changes in the water level through different adaptation strategies. Compared to that of the control group, plant growth was better with a 0–0.4 cm/d water level rate, and plant growth was in the 42–56 cm range to that a 1.0–1.4 cm/d water level rate. Furthermore, it was observed that 0–0.4 cm/d was the most suitable growth rate, with 0.6–1.0 cm/d and 0–32 cm being the ideal plant tolerance ranges, and increasing to 1.0–1.4 cm/d and 32–56 cm exceeds the plant tolerance threshold. In the middle and late period of the experiment (25–45 d), the ecological characteristics of the plants changed significantly. For example, the root-to-shoot ratio of the plant in the stable water level reached 26.1. In our field observations, plant biomass can be influenced by a variety of environmental factors. The frequency of the species was the largest at an elevation of 15 m, and the growth status of the dominant and companion species of *C. cinerascens* was weakened with an increase in soil moisture content. The suitable water content for *C. cinerascens* growth was 27.6–57.3%, the distribution elevation was 12.54–16.59 m, and the optimum elevation was 13.56–15.54 m. The study is expected to provide a reference for wetland ecology research and wetland protection and restoration, a theoretical reference for the coordination of water resource development and utilization of Poyang Lake and ecological protection of important lakes and wetlands, and an important scientific basis for wetland hydrologic regulation, ecological restoration and biodiversity conservation.

## Introduction

Water regime is one of the most important environmental factors in lake ecosystems. It plays a major role in environmental soil conditions, species distribution and vegetation composition. Any significant disruption in the hydrological regime of the lake can impair the ecological integrity of the lake and induce adverse human effects, such as eutrophication and cyanobacteria blooms^1,2^. Wetlands are diverse and productive ecosystems that harbor a variety of plants and animals and provide valuable goods and services to human society^3,4^. In the past few decades, most wetlands worldwide have been degraded to various degrees due to hydrological alteration, pollution, habitat loss, and species invasion^5,6^. China has also lost large areas of wetlands. In the Yellow River Delta, the total area of natural wetlands decreased from 2565.6 km^2^ in 1986 to 1574.5 km^2^ in 2008^7^. Decreasing water levels, caused by extensive human activities, global warming, and decreasing precipitation have also transformed large areas of marshland in China’s Sanjiang Plain into meadows^8^, and 65% of marshes in that area were lost between 1975 and 2004^9^. Among these threats, hydrological alteration is the most important because periodic hydrological processes are widely regarded as the main driving forces in riverine ecosystems^4^. Changes in the natural flow regime can also complicate the provision of ecosystem services and the sustainable use and management of standing water bodies.

In wetlands and lakes, water level fluctuations (WLFs) are very important factors that have a significant impact on the ecology, function and management of lakes^4,10,11^. The WLFs of lake ecosystems have significant temporal distribution characteristics and can be divided into two kinds of water level change modes: annual and interannual^12^, and these two variabilities can be greatly influenced by regional climatic conditions and human activities^13,14^. Therefore, numerous studies have focused on water pollution under the conditions of WLFs^15–17^. Some scholars are concerned about the impact of water level fluctuations on the overall structure, hydrological characteristics, and macrophytes of lakes and rivers^18–22^. However, the relative effects of WLFs on individual biological species and communities are less clear than the effects of other physical and chemical variables. There are few comprehensive studies on wetland plant individuals and populations under fluctuating water levels^23^.

Wetland plants are an important component of wetland ecosystems and the main primary producers. The distribution of wetland plants is affected by various environmental factors, such as topography, soil, water, and biology, and shows a certain distinct zonality^24^. This distribution shows significant temporal fluctuations in response to the hydrological regime by both direct and indirect effects^25,26^. Regarding direct effects, WLFs could not only cause a mixing disturbance^27^ but also have a strong dilution effect on aquatic plant community dynamics^28,29^. With respect to indirect effects, water level reduction could increase sediment resuspension and turbidity, decrease light availability, and finally change the plant community structure^27,28^. At the same time, plants over the course of their life can form a series of morphological characteristics and population strategies to adapt to long-term periodic WLFs variability^30–32^. For example, in lakes, the difference in the spatial distribution of plants from lake shores to lake centers is a result of the differential adaptation and response of wetland plant populations to water conditions^33–36^. The hydrographic characteristics of wetlands are closely related to the compositional, structural and distributional characteristics of plant communities. The ecological amplitude of plants reflects the relationship between plants and the environment and the ability of plants to adapt to unfavorable environments. Therefore, the ecological breadth has an important influence on the distribution of plants^37^. According to Shelford’s law of tolerance, organisms have tolerance ranges of upper and lower tolerance limits for ecological factors^38^. Previous studies have shown that the response of the ecological characteristics of wetland plants to hydrological factors typically conforms to a Gaussian distribution^39^. Therefore, the Gaussian model is the optimal model for calculating ecological amplitude^40^. Improving the knowledge base of the ecological adaptability and population characteristics of aquatic plants to changes in hydrological conditions is of great value for developing better predictions of the dynamics of aquatic communities and for the management of aquatic ecosystems in the context of climate change.

WLFs are also key ecological processes that influence plant population growth and distribution in the riparian zone^41,42^. The aquatic ecological environment changes with the pattern of water level fluctuations^43,44^. The relationship between WLFs and plant ecology has previously demonstrated that wetland plants are sensitive to large ranges of fluctuations and may in some cases be able to adapt to smaller and medium fluctuations to obtain more biomass^45–47^ In some extreme cases, the individual growth and development and population formation and development of some species are completely dependent on water level fluctuations^48,49^. The study of plant responses to fluctuations is more focused on setting different water levels to explore their effects on plant population characteristics, species diversity and life history^19,50–54^. Other studies analyzed the physiological and biochemical effects of water content or water depth gradients in different soils on plants^34,55–57^. Part of the research focuses on the tolerance characteristics of plant population structure. For example, Juan-Ovejero et al.^58^ found that the balance of tolerance to drought and saturation determined the temporal dynamics and vertical stratification of the soil invertebrate populations. Wang et al.^59^ found that WLFs may potentially increase the vegetative spread of submerged macrophyte communities, and managing WLFs may be helpful for the restoration of submerged macrophyte communities in degraded wetlands. However, there are few studies on the growth tolerance characteristics of aquatic plant populations^59^. An experiment using seed bank material from two wetland studies has shown that moderate levels of water fluctuation could promote seed germination and seedling establishment, while intensive fluctuation could greatly restrict plant growth and distribution^60^. However, there have been fewer efforts that have focused on the ecological adaptability and population characteristics of *C. cinerascens* under different water environment conditions^23,61^; in particular, the application of the Gaussian model is even rarer.

In this study, field observations and simulation experiments were combined. We examined the response of the growth process of *C. cinerascens* under WLFs and different field hydrological conditions to clarify the ecological adaptation mechanism and population characteristics in response to rhythmic hydrological changes in the Poyang Lake wetlands.

## Materials and method

### Study area and plant species

**Figure 1:**
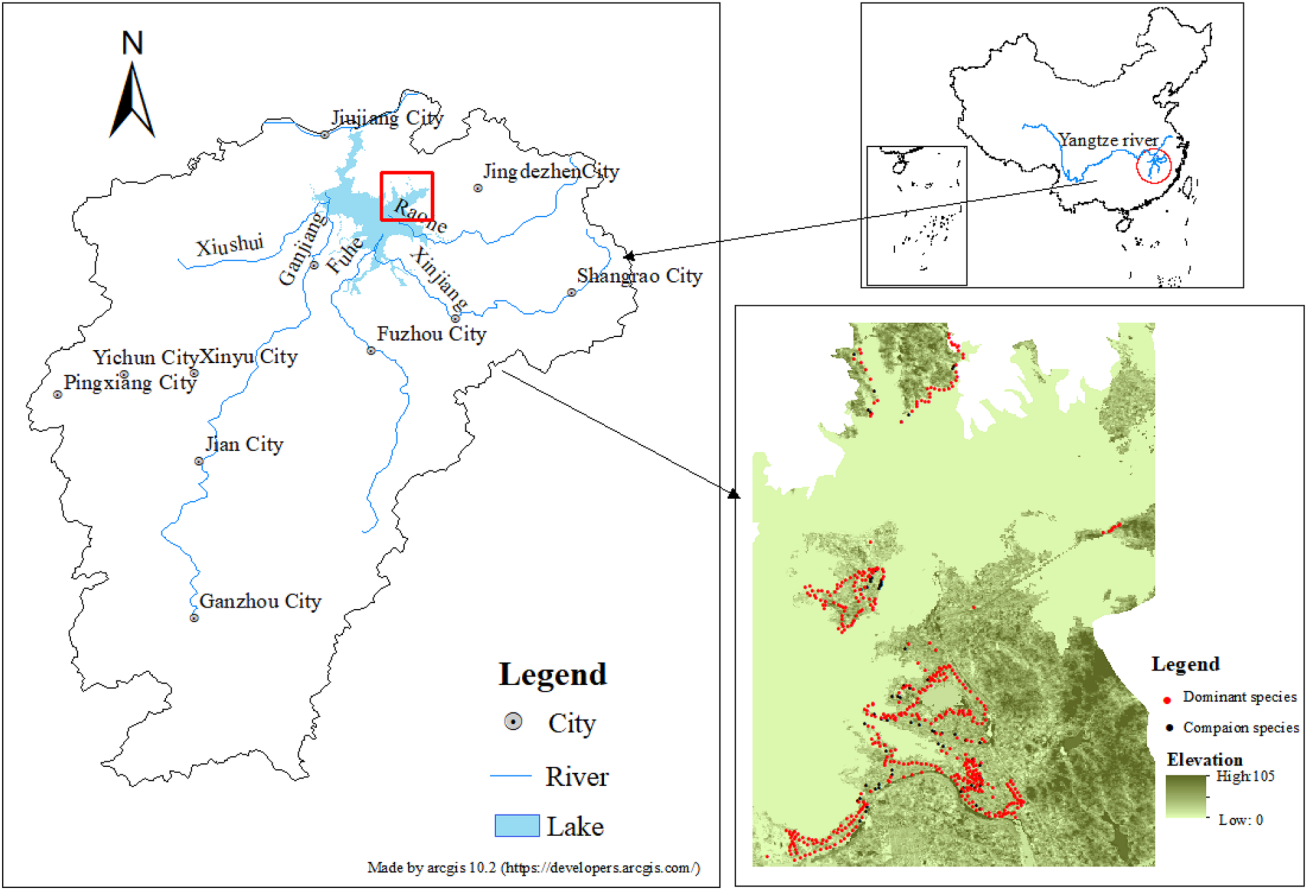
Location of Poyang lake, China and sampling point distributions.

Poyang Lake (28°22′-29°45′N, 115°47′-116°45′E) is located in the middle and lower reaches of the Yangtze River in Jiangxi Province, China (Fig. 1). It is China's largest freshwater lake and an important internationally recognized wetland. It receives water inputs mainly from five rivers, the Fuhe, Ganjiang, Raohe, Xiushui, and Xinjiang rivers, as well as a number of other minor tributaries, and discharges into the Yangtze River from a narrow outlet in Hukou. WLFs are affected by water from the five rivers and the Yangtze River. The seasonal change in the water level of the lake is obvious and often experiences a water drop of up to 10 m^62^, showing a unique hydrologic rhythm that alternates between the wet season and the dry season^63,64^. In recent years, especially since 2000, the hydrological regularity of Poyang Lake has changed significantly with global climate changes and the disturbance caused by human activities, such as the construction and operation of the Three Gorges Water Control Project. The nature of these recent changes is that the low water period is advanced and prolonged, the high-water period is shortened and the overall water level is reduced. Additionally, the daily fluctuation range of the lake water level has increased^65,66^. The dramatic WLFs changes provide a wide range of beach extents and specific hydrological and soil conditions for the growth of wetland plants, so that different plant types present irregular distribution characteristics along elevation^67^and have a significant impact on the functions of this wetland ecosystem.

*C.* *cinerascens* belongs to *Carex* of *Cyperaceae*, with rhizome and cluster perennial herbs, which are distributed in East China and central China. It is the most widely distributed dominant species in Poyang Lake, covering an area of 960 km^2^ and accounting for 28.8% of the water surface area of Poyang Lake, and it plays an important role in the function of the Poyang Lake wetland ecosystem^68^.

### Experimental design

#### Field investigations

A field investigation of Baishazhou National Wetland Park was carried out in the dry season of Poyang Lake during November 2016. The analyses were carried out by considering the plant species, growth status, coverage, height and soil moisture status in each study area through the combination of a sample point method and a sample line method. Photos were taken of each sample point, GPS positioning was employed, plant samples were collected, comparisons were made with imaged indoor plants^69^ for identification of plant species, and the elevations of various points were obtained according to longitude and latitude.

#### Simulation experiment

The laboratory experiment was carried out by the two-set basin method. The experimental site was the plant sunlight room of the Key Laboratory of Poyang Lake Wetland and Watershed Research Ministry of Education, Jiangxi Normal University, using a temperature of 22 ± 5 °C and natural light. On March 15, 2019, seedlings of *C. cinerascens* were collected from the typical distribution area in Nanji Wetland National Nature Reserve (28°55′45″N, 116°19′26″E) and then cultivated in a 17 cm × 18 cm pot for preculture, with ten plants per pot. The substrate was a meadow marsh soil collected from the Poyang Lake wetland (pH 5.36, organic matter content was 42 g/kg, and total nitrogen content was 19.6 g/kg). On April 10, plants with the same height (74.6 cm) were placed in the plexiglass bucket used for the experiment. Each glass barrel had a small hole with a diameter of 4 cm at a distance of 10 cm from the bottom, sealed with a rubber plug, which could be used to adjust the height of the water level in the glass barrel.

The test was from April 10 to May 20, 2019, for a total of 40 d. Three types of water level were used, the rising water level, the falling water level and the stable water level, which were set with seven water level gradients, 21 test groups and a 0-cm water level gradient (the water level does not pass the surface of the culture medium in the culture basin) control group. The test adopts a random area group setting with three repetitions for each group setting. The initial water levels of the stabilized fluctuations and falling water levels were 8 cm, 16 cm, 24 cm, 32 cm, 40 cm, 48 cm, and 56 cm. The initial water level gradient of the rising fluctuation was 0 cm, and the rise and fall rates were 0.2 cm/d, 0.4 cm/d, 0.6 cm/d, 0.8 cm/d, 1.0 cm/d, 1.2 cm/d, and 1.4 cm/d. Water levels were tested at 19:00 daily.

### Data collection

In the field investigation portion of our study, the plant coverage was measured by the detection-visualized method, the community height was measured by tape measure, and the soil volume moisture content was measured and classified by the HH2 instrument according to the following categories: < 10% (dry), 10–30% (slightly wet), 30–50% (very wet), 50–70% (saturated), and > 70% (flooded)^70^. According to the soil volume moisture content, different weights were given to each type of soil moisture status, among which dry was 1, slightly wet was 2, very wet was 4, saturated was 6, and flooded was 8.5^70^.

Experimental indicators were measured once as a starting reference value before the water level fluctuated (10 April). Plant height was the highest plant in the same experimental treatment group measured by a tape measure every five days. The biomass of each group was measured, and the average biomass of each plant was calculated at 0, 25 and 45 days. The measured value after the end of the water level fluctuations of each test indicator (May 25) was taken as the final value.

The distribution of biomass was reflected by the root shoot ratio, and the accumulation of biomass was reflected by the absolute growth rate (AGR) and relative growth rate (RGR) in the biomass growth rate analysis. The calculation formulas are as follows:

1 $${\text{AGR}} = \frac{{B_{i + 1} - B_{i} }}{{t_{i + 1} - t_{i} }}$$

2 $${\text{RGR}} = \frac{{\ln B_{i + 1} - \ln B_{i} }}{{t_{i + 1} - t_{i} }}$$

where B_i_ and B_i + 1_ is the biomass of the *Carex cinerascens* population at T_i_ and T_i + 1_, respectively.

### Data analysis

The frequency of *C. cinerascens* in the elevation zone was analyzed using a Gaussian model to obtain the relationship between the growth of *C· cinerascens* and elevation^71^:

3 $$y = y_{0} + Aexp[ - 0.5\left( {x - x_{c} )^{2} /w^{2} } \right]$$

Where *y* represents the index of the biological or ecological characteristics of the species, *A* + *y*_*0*_ represents the maximum value of the corresponding index, *x*_*c*_ represents the minimum value of certain environmental factors to plant species when the corresponding biological indicator reaches the maximum value, and *w* indicates the resistance of the species, which can also be described as the amplitude of the species. In general, suitable ecological amplitude is [*x*_*c*_*-2w, x*_*c*_ + *2w*], and optimum ecological amplitude is [*x*_*c*_*-w, x*_*c*_ + *w*].

Experimental data were processed using Excel 2010 and Origin 2018 for data processing and drawing, the overview map of the study area was produced through arcgis 10.2. The statistical analysis of the measured data was carried out with SPSS 22.0. The least significant difference method was used to test the difference between the data. The repeated measurement variance method was used to analyze the relationship between *C. cinerascens* treatments in different time periods; before analysis, a sphere test was performed on repeated measurement data. If the test result was *p* > 0.05, the single-factor analysis of variance method was used; if the test result was *p* < 0.05, the multivariate analysis of variance method was used to process the data.

## Results

### Plant height

The variation trend of plant height was significantly different at different water levels during the experiment. When the experiment lasted for 20 days, the differences in plant height under different treatment intensities across three types of water levels were more obvious. In a stable water environment, for the water level range of 0–32 cm, the plant height increased with time, and the plant height increased with an increase in flooding depth. When the water level was greater than 40 cm, the plant height decreased with time. Throughout the stable water level experiment, the maximum increase in plant height occurred in the control group (56.46%), and the maximum decrease in plant height appeared at a flood depth of 56 cm (49.17%). In the WLFs test group, rates of water level change in the range of 0–0.6 cm/d induced an upward trend in plant height over time. For the range of 0.8–1.4 cm/d, the rising water level group first grew and then diminished, while the falling water level treatment group first decreased and then increased growth, and after 20 days, the plant height of the control group was higher than that of the other experimental groups. In both cases, the trend of plant height changed within a time range of 15–25 days. For the rising water level cases, the plant height increases in the 0.2 cm/d and 0.4 cm/d treatment groups were significantly higher than that of the control group (* p* < 0.05), and the plant height of the 0.2 cm/d group was higher than that of the other experimental groups at 25–45 days. (Table 1).

**Table 1:** Effect of different hydrological environments on the plant height of *C. cinerascens* based on laboratory simulation experiments.

	0 cm·d^−1^	0.2 cm·d^−1^	0.4 cm·d^−1^	0.6 cm·d^−1^	0.8 cm·d^−1^	1.0 cm·d^−1^	1.2 cm·d^−1^	1.4 cm·d^−1^
	0 cm	8 cm	16 cm	24 cm	32 cm	40 cm	48 cm	56 cm
**Rising water level**								
0d	74.57 ± 16.97	74.57 ± 16.97	74.57 ± 16.97	74.57 ± 16.97	74.57 ± 16.97	74.57 ± 16.97	74.57 ± 16.97	74.57 ± 16.97
5d	75.73 ± 13.34	75.90 ± 13.10	79.30 ± 11.20	76.30 ± 12.64	75.80 ± 8.88	77.40 ± 16.60	81.60 ± 16.77	83.60 ± 14.45
10d	80.23 ± 15.10	81.60 ± 12.20	86.50 ± 13.50	77.90 ± 12.64	76.40 ± 15.78	82.20 ± 15.40	84.60 ± 12.43	84.80 ± 13.65
15d	88.60 ± 11.05	86.90 ± 15.40	88.10 ± 9.80	79.10 ± 12.64	78.30 ± 13.56	85.80 ± 20.70	85.10 ± 18.76	80.70 ± 10.76
20d	106.80 ± 23.06	96.40 ± 13.60	93.20 ± 13.30	83.20 ± 12.64	84.50 ± 17.80	88.40 ± 7.80	80.80 ± 13.76	72.20 ± 11.32
25d	109.20 ± 22.13	121.80 ± 19.70	107.90 ± 14.10	93.40 ± 12.64	88.60 ± 16.50	103.60 ± 9.60	74.20 ± 4.67	63.20 ± 9.65
30d	113.50 ± 20.43	122.50 ± 18.50	117.60 ± 19.00	97.80 ± 12.64	91.40 ± 20.40	94.60 ± 11.50	70.10 ± 7.34	60.30 ± 7.45
35d	107.67 ± 16.10	125.40 ± 21.10	123.50 ± 26.70	104.30 ± 12.64	87.40 ± 19.80	89.30 ± 14.10	68.60 ± 9.45	58.90 ± 8.46
40d	114.20 ± 24.60	125.80 ± 24.60	119.20 ± 17.80	105.50 ± 12.64	83.30 ± 17.70	80.70 ± 12.78	67.80 ± 8.21	58.10 ± 6.65
45d	116.67 ± 26.70	124.90 ± 26.80	117.80 ± 21.19	108.60 ± 12.64	80.90 ± 10.90	77.10 ± 11.76	64.20 ± 8.87	56.30 ± 5.89
**Falling water level**								
0d	74.57 ± 16.97	74.57 ± 16.97	74.57 ± 16.97	74.57 ± 16.97	74.57 ± 16.97	74.57 ± 16.97	74.57 ± 16.97	74.57 ± 16.97
5d	75.73 ± 13.34	76.80 ± 11.30	78.40 ± 9.50	79.60 ± 9.06	76.50 ± 14.50	73.90 ± 8.90	65.40 ± 8.76	67.70 ± 10.23
10d	80.23 ± 15.10	87.20 ± 12.60	83.10 ± 10.80	83.60 ± 9.06	75.80 ± 16.70	70.60 ± 7.80	58.30 ± 6.78	42.90 ± 8.88
15d	88.60 ± 11.05	90.80 ± 13.60	85.40 ± 12.50	82.20 ± 9.06	71.30 ± 12.10	68.80 ± 10.10	44.50 ± 4.23	41.10 ± 6.56
20d	106.80 ± 23.06	95.60 ± 14.10	78.40 ± 13.70	79.20 ± 9.06	73.80 ± 9.67	68.20 ± 6.66	46.70 ± 5.43	43.20 ± 5.87
25d	109.20 ± 22.13	107.20 ± 19.30	97.10 ± 8.90	95.30 ± 9.06	77.30 ± 10.60	58.90 ± 7.67	54.30 ± 8.46	48.70 ± 9.46
30d	113.50 ± 20.43	109.20 ± 23.10	98.20 ± 14.60	95.80 ± 9.06	81.70 ± 16.50	63.50 ± 12.34	64.70 ± 10.46	54.90 ± 7.25
35d	107.67 ± 16.10	97.40 ± 20.50	97.20 ± 20.10	96.10 ± 9.06	83.20 ± 12.82	72.10 ± 13.09	71.80 ± 8.67	67.90 ± 8.81
40d	114.20 ± 24.60	98.40 ± 12.60	96.90 ± 16.60	96.90 ± 9.06	84.20 ± 11.20	70.30 ± 10.18	68.50 ± 9.93	54.30 ± 4.44
45d	116.67 ± 26.70	87.10 ± 17.60	96.30 ± 15.90	97.90 ± 9.06	85.60 ± 15.30	67.20 ± 7.87	56.80 ± 11.79	48.30 ± 5.54
**Static water level**								
0d	74.57 ± 16.97	74.57 ± 16.97	74.57 ± 16.97	74.57 ± 16.97	74.57 ± 16.97	74.57 ± 16.97	74.57 ± 16.97	74.57 ± 16.97
5d	75.73 ± 13.34	76.00 ± 15.60	77.20 ± 6.36	70.40 ± 6.31	73.20 ± 11.10	69.80 ± 9.69	71.70 ± 9.34	67.50 ± 8.78
10d	80.23 ± 15.10	80.40 ± 11.60	82.80 ± 7.90	78.90 ± 13.40	66.60 ± 10.00	64.90 ± 9.10	53.40 ± 3.20	49.80 ± 5.63
15d	88.60 ± 11.05	86.10 ± 14.30	84.70 ± 11.10	80.10 ± 21.60	68.60 ± 10.40	68.60 ± 7.60	48.60 ± 6.66	45.30 ± 6.98
20d	106.80 ± 23.06	93.60 ± 17.80	76.30 ± 12.40	82.80 ± 18.80	75.20 ± 9.80	64.10 ± 6.80	46.90 ± 5.40	43.20 ± 4.53
25d	109.20 ± 22.13	107.80 ± 19.70	87.40 ± 8.90	83.40 ± 16.50	94.10 ± 16.70	51.20 ± 6.69	44.60 ± 7.30	41.30 ± 3.47
30d	113.50 ± 20.43	110.30 ± 14.34	89.10 ± 21.10	85.80 ± 17.60	98.60 ± 21.11	49.20 ± 10.34	46.90 ± 6.70	41.50 ± 6.53
35d	107.67 ± 16.10	105.80 ± 20.80	91.80 ± 23.50	88.40 ± 12.90	94.50 ± 15.76	48.60 ± 11.45	52.10 ± 9.97	40.30 ± 5.98
40d	114.20 ± 24.60	106.80 ± 20.60	89.60 ± 17.80	88.60 ± 16.88	94.80 ± 18.67	50.10 ± 15.65	46.20 ± 11.36	38.90 ± 3.05
45d	116.67 ± 26.70	106.20 ± 19.90	90.30 ± 18.90	89.40 ± 16.00	95.10 ± 12.85	60.30 ± 13.76	41.30 ± 11.67	37.90 ± 4.76

### Biomass allocation and accumulation

#### Biomass allocation

In different water environment types, plant growth for different experimental periods had different trends. The total biomass and aboveground biomass of *C. cinerascens* increased continuously with time at a low- and medium-strength water level rate (0–1.0 cm/d, 0–40 cm) and changed and decreased with time for a high-strength water level rate (1.2–1.4 cm/d, 48–56 cm). For the stable water level experimental runs, the underground biomass of the 0–32 cm experimental groups showed a continuous increase; in the 40 cm flooding experiment, the biomass continued to increase. The aboveground biomass also decreased with time for a flood level of 40 cm. For submergence depths of 40 cm and 56 cm and a stable water level, the biomass trend first increased and then decreased. At the end of the experiment, the underground biomass of each group was significantly higher than the initial control value (0 d, 0 cm) (*p* < 0.05). The maximum increase in underground biomass was 29.67% (rising water level rate of 0.2 cm/d), and the minimum increase was 2.71% (falling water level rate of 1.0 cm/d). During the whole experimental period, the maximum aboveground biomass of the three water level types was 0.3896 g (rising water level, 0.2 cm/d), and the maximum total biomass was 1.1134 g (rising water level, 0.2 cm/d).

**Figure 2:**
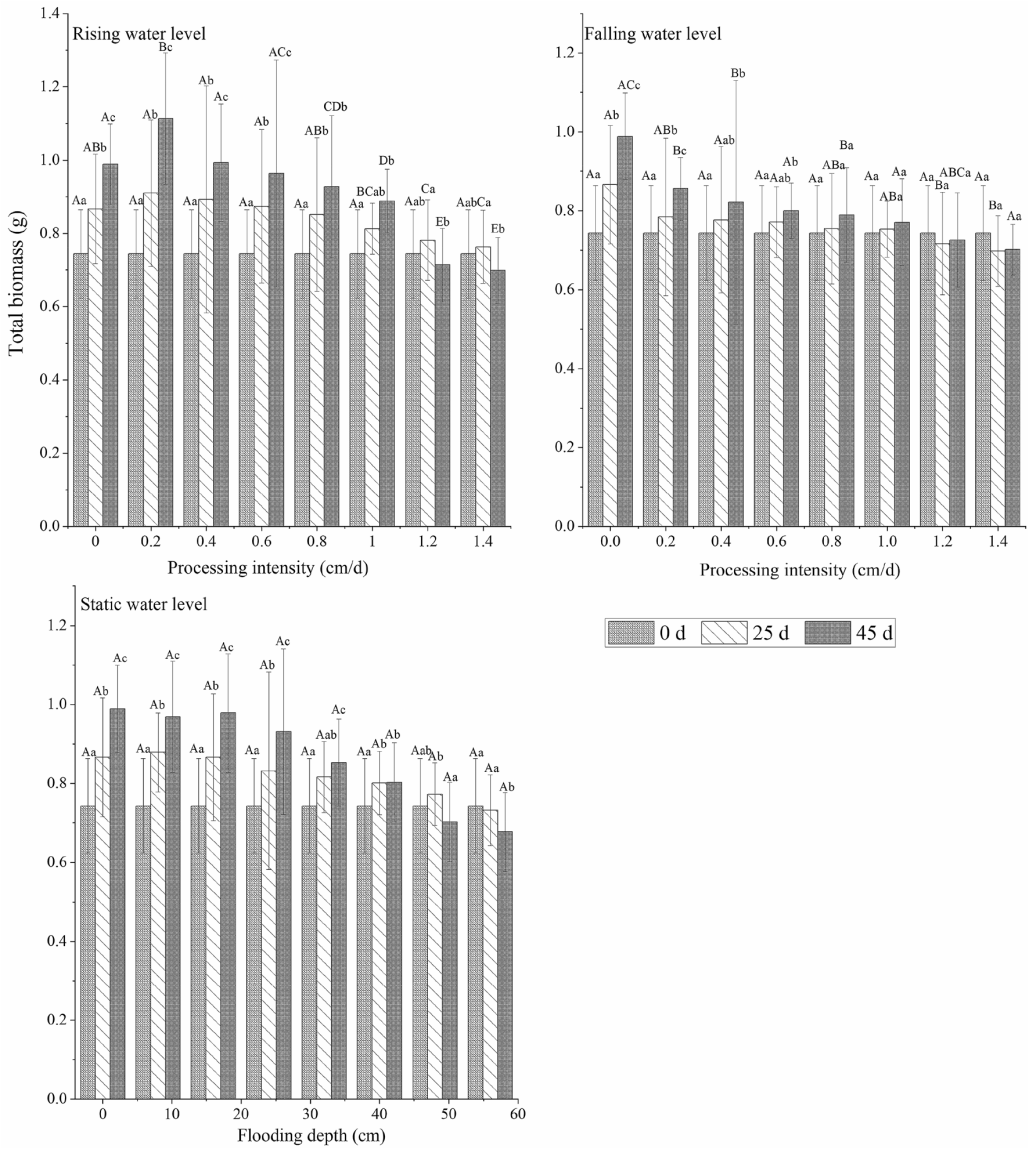
Effects of different hydrological environments on the total biomass and bioavailability of *C. cinerascens.*

**Figure 3:**
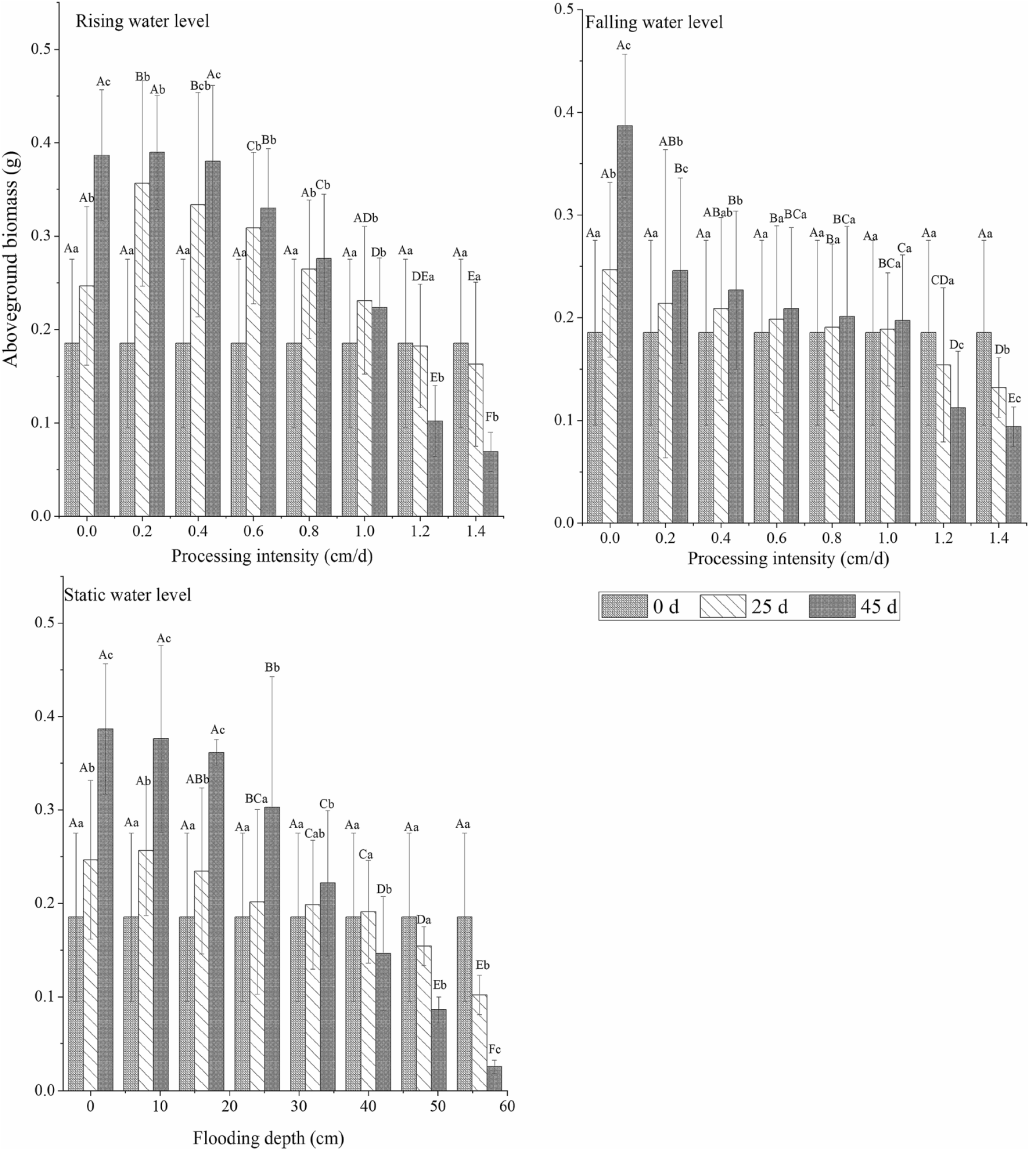
Effects of different hydrological environments on aboveground biomass and bioavailability of *C. cinerascens.*

**Figure 4:**
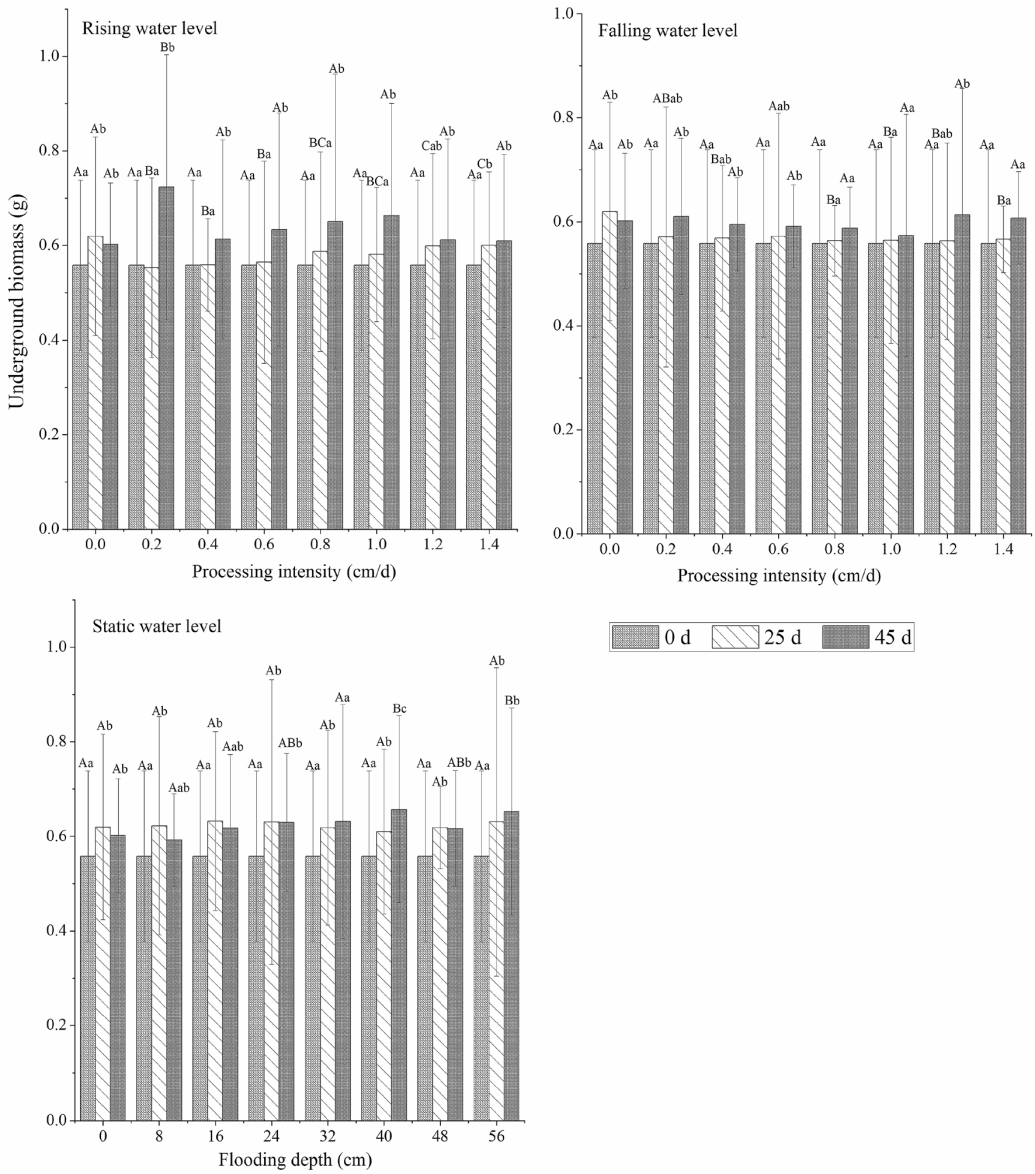
Effects of different hydrological environments on underground biomass and bioavailability of *C. cinerascens.*

**Figure 5:**
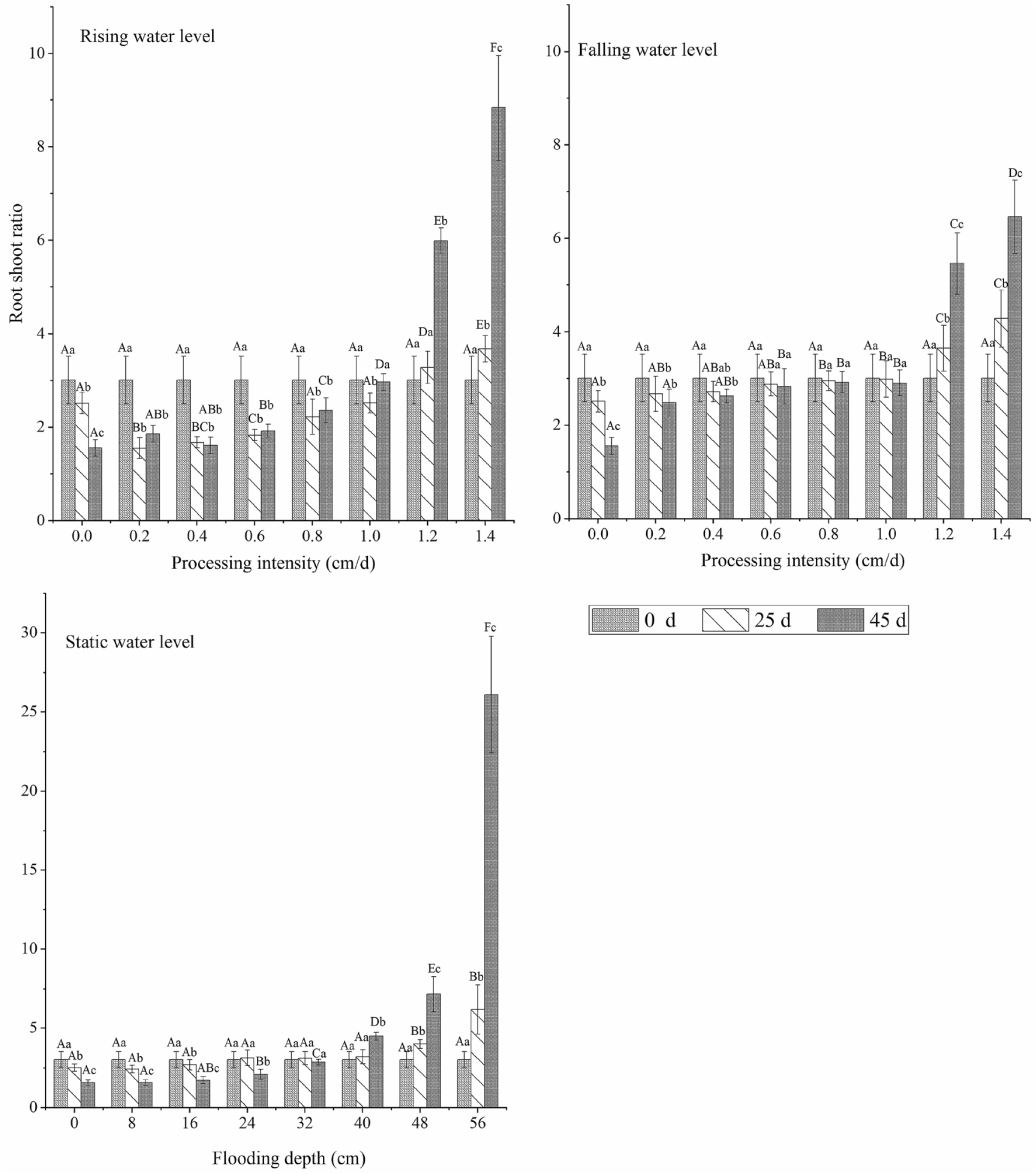
Effects of different hydrological environments on the root-to-shoot ratio of biomass and bioavailability of *C. cinerascens.*

The change in time of the root-to-shoot ratio of *C. cinerascens* in different water environment types was roughly opposite the change in aboveground biomass. The root-to-shoot ratio of plants in the three water level environments was higher than that in other experimental groups. The root-to-shoot ratio increased continuously with time in the experimental treatment group, with a water level rate of change of 1.2–1.4 cm/d and a stable water level depth of 40–56 cm, and it decreased in the other experimental groups. In the later period of the experiment (25–45 days), the root-to-shoot ratio slightly increased or remained relatively stable. Over time, the largest reduction was in the control group, and the largest increase was in the 1.4 cm/d and 56 cm treatment groups. When the stable water level was 56 cm at the end of the experiment, the root-to-shoot ratio was the highest (26.10). (Figs. 2, 3, 4, 5).

#### Biomass accumulation

**Figure 6:**
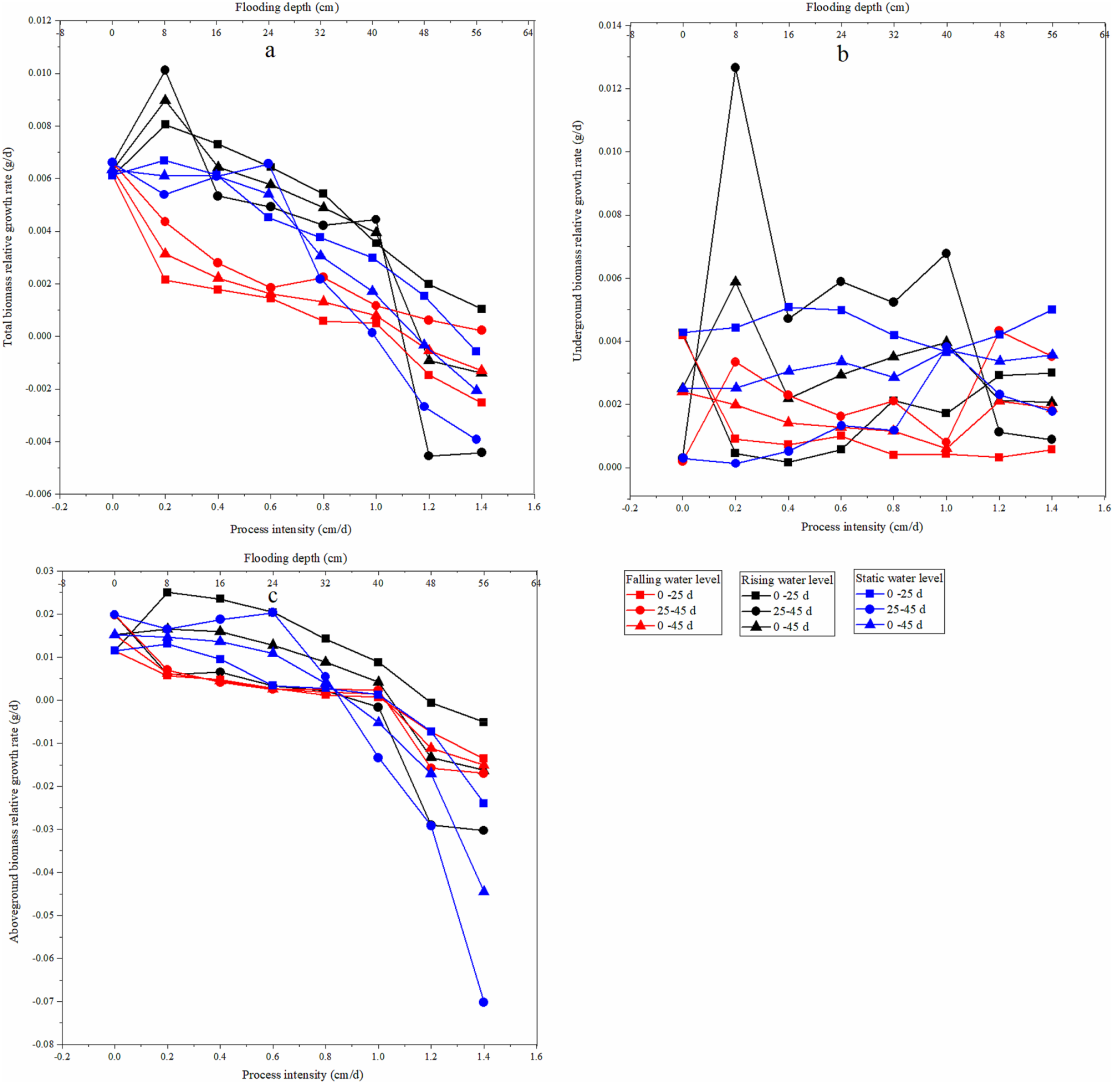
Effect of different hydrological environments on the relative growth rate of the biomass of *C. cinerascens.* (**a**) Total biomass relative growth rate. (**b)** Underground biomass relative rate. (**c)** aboveground biomass growth rate.

**Figure 7:**
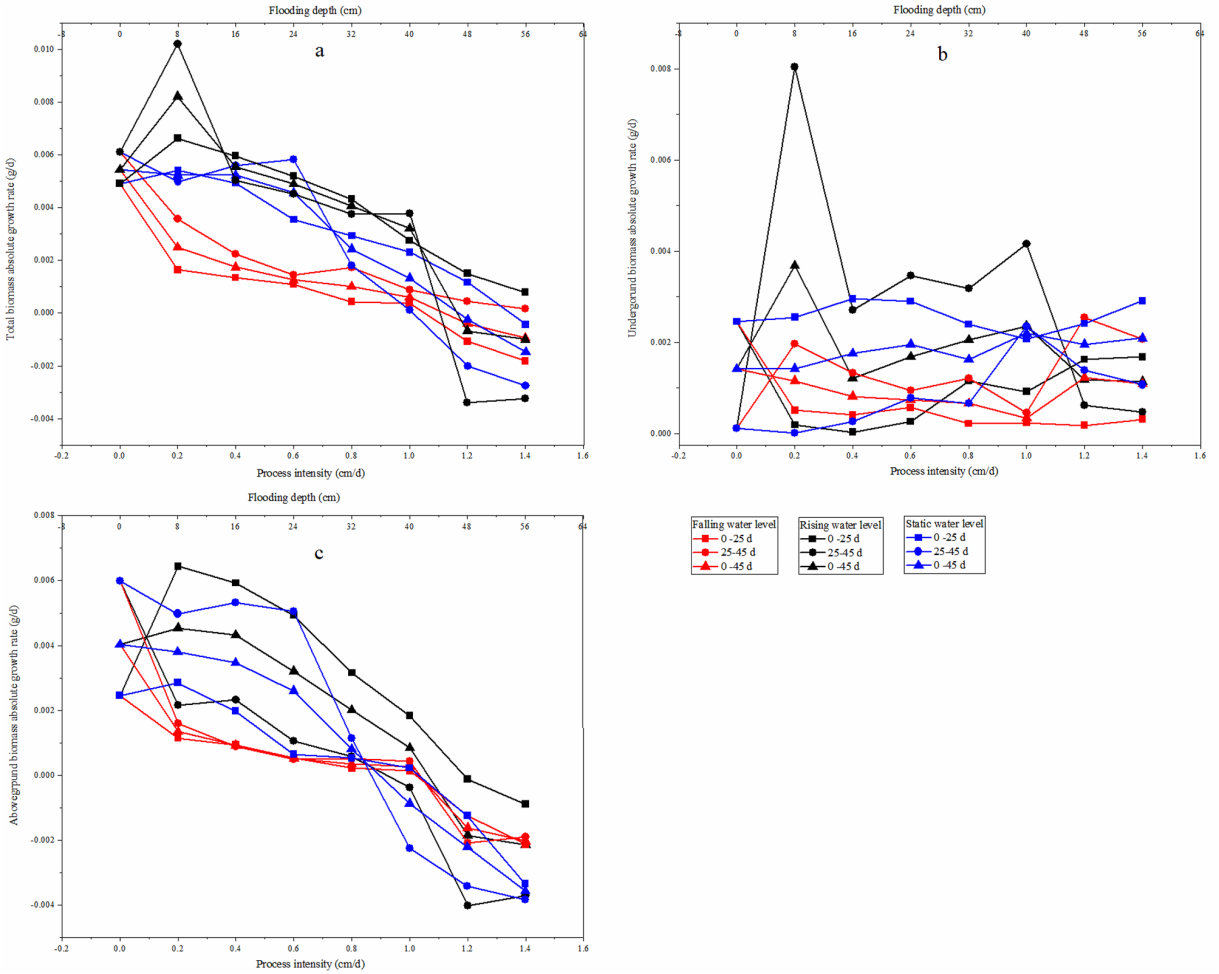
Effect of different hydrological environments on the absolute growth rate of the biomass of *C. cinerascens.* (**a**) Total biomass absolute growth rate. (**b**) underground biomass absolute rate. (**c**) aboveground biomass absolute rate.

The biomass accumulation of *C. cinerascens* in different water environments highly varied in different experimental stages. The AGR of total biomass under each water environment type decreased with increasing experimental treatment intensity. The AGR of aboveground and underground biomass in the stable water level group and in the rising water level group decreased from the early stage to the later stage of the experiment, while the AGR of aboveground biomass in the falling water level treatment group had no significant change (*p* > 0.05). At the fluctuating water level, except for the rising fluctuation of 1.2–1.4 cm/d, the underground biomass of the other experimental groups decreased from the early stage to the later stage. From the RGR, the underground biomass of *C. cinerascens* in different water level environments was always in the accumulation process, while the accumulation of total biomass and aboveground biomass in the WLFs group with 1.2–1.4 cm/d and 48–56 cm was blocked (Figs. 6, 7).

### Field distribution characteristics of the *Carex* population

**Figure 8:**
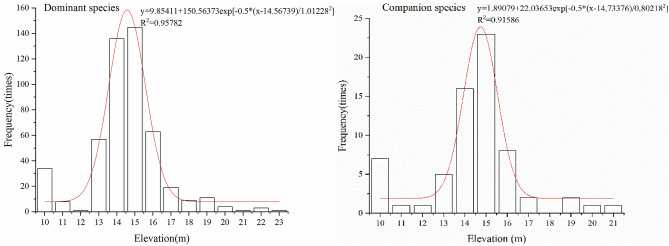
Gaussian population distribution characteristics of *C. cinerascens* at different elevations.

A total of 815 sample points and 56 species of plants were recorded in the field survey, of which 492 were dominant species, accounting for 60.37% of the sample points, and 67 were companion species, accounting for 8.22% (Fig. 1). The growth and distribution of the dominant species and companion species of *C. cinerascens* in the field are observed to have the same trend as the elevation changes, and they are mainly distributed in an area with an elevation of approximately 15 m. The number of dominant species in the range of 14–16 m elevation is as high as 344, and that of companion species is 47 (Fig. 8). The Gaussian regression analysis shows that a suitable distribution of *C. cinerascens* is in the elevation range of 12.54–16.59 m (Table 2).

**Table 2:** Gaussian statistics of the elevation range of distuributed *C. cinerascens.*

Project	Optimum distribution range/m	Suitable distribution range/m
	[X_C_-W, X_C_ + W]	[X_C_-2 W, X_C_ + 2 W]
Dominant species	[13.56, 15.80]	[12.54, 16.59]
Companion species	[13.93, 15.54]	[13.13, 16.34]
Intersection	[13.56, 15.54]	[12.54, 16.59]

### Effect of soil water conditions on the growth of the *Carex* population

**Figure 9:**
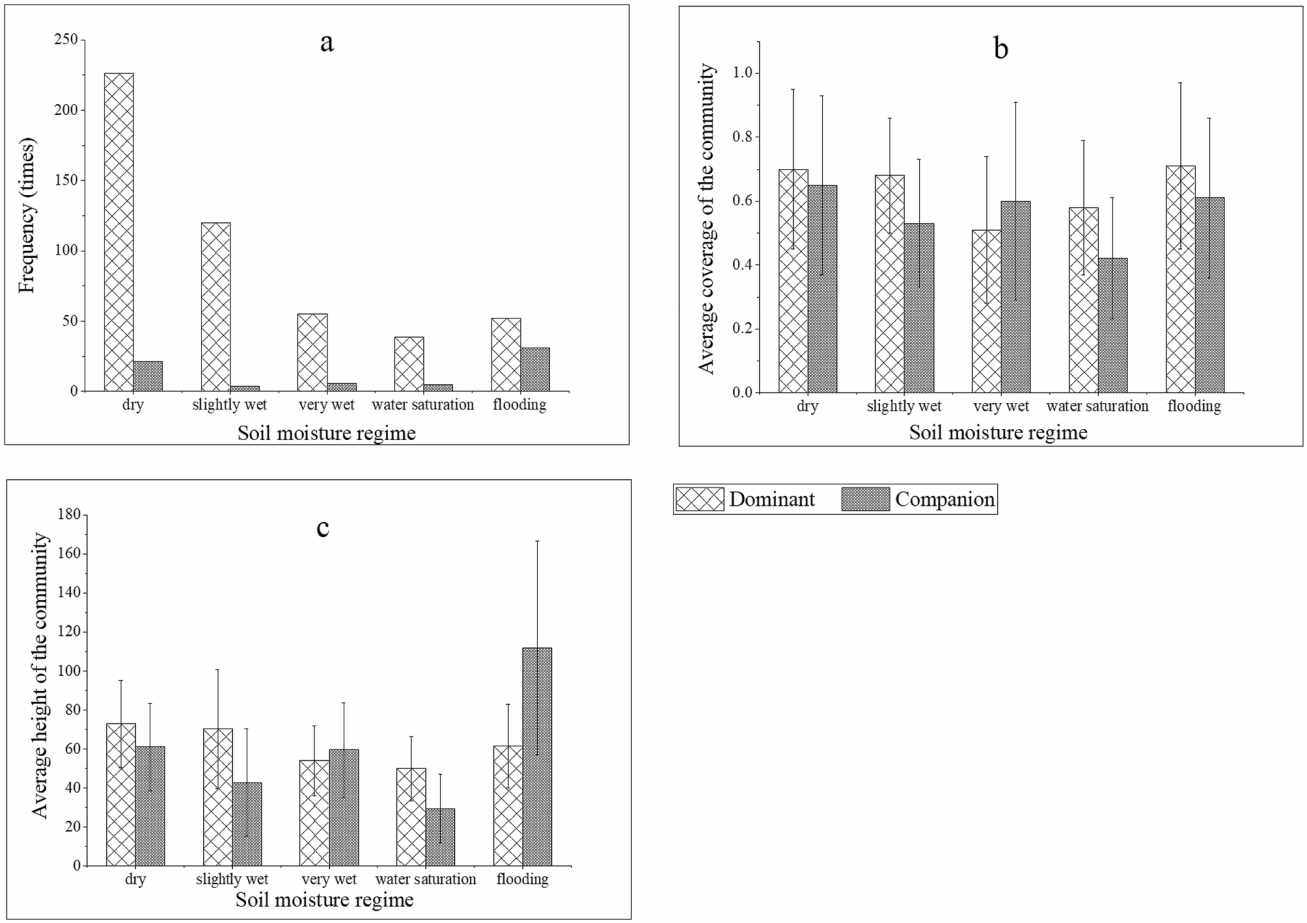
Characteristics of *C. cinerascens* population changes with soil moisture regime. (**a**) Frequency of the *C. cinerascens* population. (**b**) Coverage of the *C. cinerascens* population. (**c**) Average height of the *C. cinerascens* population.

**Figure 10:**
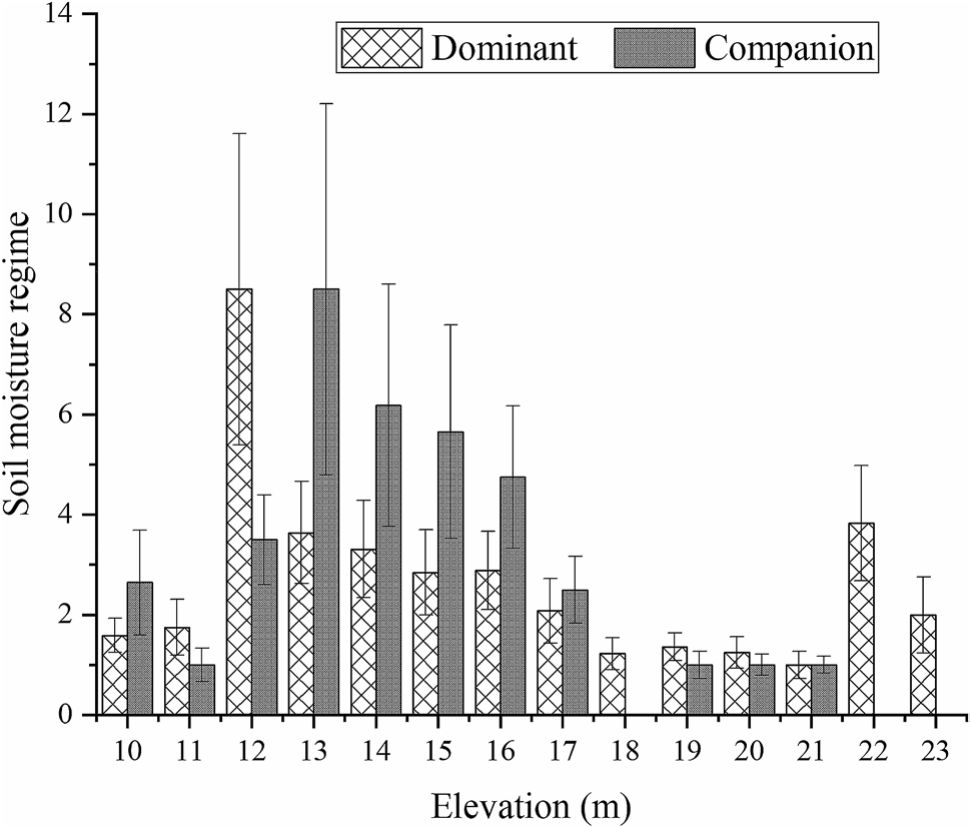
Changes in soil water weight with elevation of the *C. cinerascens* population.

The growth characteristics of the dominant species and companion species of *C. cinerascens* populations under different soil water conditions varied. The species community was mainly distributed in areas of soil moisture content less than 10% and soil moisture weights of 3.5–8.5 in the field environment. Conditions of 30% soil moisture as well as flood environments showed community growth that was better than that for conditions of 30–70% soil moisture, and there was no significant difference between the community coverage and height for dry, slightly wet and flooded conditions (*p* > 0.05). Conversely, for very wet and saturated conditions, the difference was significant (*p* < 0.05). Additionally, the growth of the companion species of *C. cinerascens* was similar to that of the dominant species in different soil moisture content environments (Figs. 9, 10).

## Discussion

### Adaptability of the growth of *C. cinerascens* to environmental water level changes

The growth conditions, such as plant height, stem length and leaf shape, of the aboveground sections of hygrophytes are the most direct response to changes in water level. To adapt to various conditions of WLFs, wetland plants usually make corresponding adjustments to maintain their own survival and growth^71,72^. The ability and strategy of plants to adapt to these environmental changes determine their proliferation and distribution range. An adjustment of biomass allocation is a common way for many wetland plants to adapt to changes in water level^73^. Plants living in changing habitats have adaptive strategies for the plasticity of biomass, and the results of this self-regulation are often in line with the predictions of the optimal allocation theory^74^, i.e., plants can utilize limited resources of light, nutrition and water by adjusting the biomass allocation of various organs to respond to changes in environmental conditions^75^. In the long-term adaptive evolution process, wetland plants have formed special survival strategies to reduce the impact of WLFs on plants. The most important thing is to respond to changes in the water environment through the fast-growing escape strategy and dormant, slow-growing tolerance strategy in the above-ground part^76,77^.

The results of our study determine that *C. cinerascens* has different survival strategies at different water levels. In water level environments of 1.2 cm/d, 1.4 cm/d and 40–56 cm, the oxygen supply and light in the water body are insufficient, photosynthesis is restricted, leaves wither and plants die. Furthermore, these conditions lead to a decrease in plant height, total biomass and aboveground biomass. The biomass is mainly concentrated in the underground components of the plant (Fig. 3). Zhang et al.^77^. found that the growth and photosynthesis of *Carex schmidtii* under drying treatment and reflooding treatments also decreased to different degrees. That is, the tolerance mechanism of the plant into a dormant state changes the life cycle of the life cycle strategy to respond to changes in the water environment to increase survival^78,79^. The growth and development process of plants for WLFs rates of 0.2 cm/d and 0.4 cm/d was significantly faster than that observed in the constant water level control group, since the water level was always lower than 8 cm and 16 cm, respectively, and the plants had time to adapt to each flooding depth. Thus, they could benefit from the resource allocation process before each water level change^80^. For a rising water level, *C. cinerascens* increased growth with an increase in water level; plant height, root-to-shoot ratio and biomass, exhibited varied trends over time (Figs. 2, 3, 4, 5, 6, 7, 8). The laboratory results, combined with field observations of plants and biomass in the wet and dry seasons, revealed that *C. cinerascens* can adopt an escape strategy for this intensity range and can adapt to changes in the water level environment through tolerance strategies. For a falling water level, the plant was affected by the initial water level height and the rate of WLFs. In the early and later periods of the experiment, the adaptive strategy was not utilized in the rising water level cases. Compared with rising water levels, plants with lower water levels at the same water level fluctuation rate have worse growth and development. Over time, the biomass of *C. cinerascens* began to gradually shift to the underground sections of the plant, resulting in a gradual slowing of plant height growth and the accumulation rate of aboveground biomass, while the accumulation of the underground biomass increased (Figs. 7, 8). Thus, *C. cinerascens* adopted different adaptation strategies in response to different periodic and rhythmic water level changes.

### Tolerance characteristics of the *C. cinerascens* population in response to WLFs

Various wetland plants have a specific water environment niche suitable for growth^81^, and different wetland plants have different hydrological requirements during growth and development. The frequency of flooding and fluctuation are important factors affecting the growth, reproduction and population distribution of wetland plants^21,47^. However, field observations reveal that there are many environmental factors that may affect plant populations. In addition to hydrological factors, soil nutrients, temperature, precipitation and intraspecific and interspecific relationships of plants will also have an impact, and the ecological characteristics of wetland plants are the result of multiple factors^82^. In the indoor simulation experiment, the ecological indexes of *C. cinerascens* showed no obvious Gaussian distribution model under different water level conditions. This may be caused by the large difference between the water level gradient set in this experiment and the water level tolerance range of *C. cinerascens.* Gaussian regression is an effective method to study the distribution characteristics of plant populations along an environmental gradient^83^. In this study, the relationship between the population distribution and elevation of *C. cinerascens* in the wild environment is reflected.

The ecological amplitude of the water level is larger for flooding and drought conditions^26^. The growth and development of plants were severely inhibited at 42–56 cm, especially in the range of 20–45 days, as the total biomass decreased significantly, and the biomass accumulation was much lower than that in the first 20 days (Figs. 3, 4, 5, 6, 7, 8). It was observed that plants can accumulate more biomass in a WLFs of 0–0.4 cm/d. For a WLFs of 0.6–1.0 cm/d, the indicators gradually began to show differences, indicating that plants were beginning to adapt to the changing water level for this range. However, field investigations showed that due to the uncertainty of the WLFs of Poyang Lake, the time of beach exposure varies, which makes the beach grow different plant communities at different elevations. However, due to the change in the hydrological situation of Poyang Lake in recent years, the growth range of *C. cinerascens* has changed from 15–17 m^84,85^ to 11 m. This result is similar to Qi et al.’s^86^ observation that water depth affects the development and expansion of *Carex appendiculate*.

## Conclusion

There is an apparent threshold water level observed that allows better growth and development of *C. cinerascens*. For a stable water level, the tolerance range of plants was 0–32 cm, and the extreme growth tolerance was 32 cm. Under changing water level conditions, 0–0.4 cm/d was the optimal rate for healthy plant growth, and 0.6–1.0 cm/d was the fluctuation range of plant growth tolerance; 1.0–1.4 cm/d was over the tolerance threshold of plants, and the plant could not grow normally and complete its life cycle. In the field environment, the population distribution of *C. cinerascens* showed typical zonal distribution characteristics, concentrated in the elevation range of 13.56–15.54 m and soil moisture of 3.5–8.5. Therefore, regulating the fluctuation of water levels can be used as a means of ecosystem management, guiding the protection and restoration of *C. cinerascens* and other wetland plants, as well as regulating the plant abundance in a wetland ecosystem. The results of our study should be of great significance to improve the research knowledge and methods of wetland hydroecology.

## References

[1] Bakker ES, Hilt S (2016). Impact of water-level fluctuations on cyanobacterial blooms: options for management. Aquat. Ecol..

[2] Li Q (2017). Impact of water level fluctuations on the development of phytoplankton in a large subtropical reservoir: implications for the management of cyanobacteria. Environ. Sci. Pollut. R..

[3] Sellheim KL, Vaghti M, Merz JE (2016). Vegetation recruitment in an enhanced floodplain: ancillary benefits of salmonid habitat enhancement. Limnol. Ecol. Manag. Inland Waters.

[4] Yuan S, Yang Z, Liu X, Wang H (2017). Key parameters of water level fluctuations determining the distribution of *Carex* in shallow lakes. Wetlands.

[5] Tharme RE (2003). A global perspective on environmental flow assessment: emerging trends in the development and application of environmental flow methodologies for rivers. River Res. Appl..

[6] Wang M, Liu Z, Luo F, Lei G, Li H (2016). Do amplitudes of water level fluctuations affect the growth and community structure of submerged macrophytes. PLoS ONE.

[7] Wang M, Qi S, Zhang X (2011). Wetland loss and degradation in the Yellow River Delta, Shandong Province of China. Environ. Earth Sci..

[8] Zhou D, Gong H, Wang Y, Khan S, Zhao K (2008). Driving Forces for the Marsh Wetland Degradation in the Honghe National Nature Reserve in Sanjiang Plain Northeast China. Environ. Model. Assess..

[9] Zhang J, Ma K, Fu B (2009). Wetland loss under the impact of agricultural development in the Sanjiang Plain NE China. Environ. Monit. Assess..

[10] Coops H, Beklioglu M, Crisman TL (2003). The role of water-level fluctuations in shallow lake ecosystems – workshop conclusions. Hydrobiologia.

[11] Leira M, Cantonati M (2008). Effects of water-level fluctuations on lakes: an annotated bibliography. Ecol. Effect Water-Level Fluctuat. Lakes.

[12] Cooke GD, Welch EB, Peterson S (1994). Restoration and management of lakes and reservoirs.

[13] Blindow I (2010). Long- and short-term dynamics of submerged macrophytes in two shallow eutrophic lakes. Freshwater Biol..

[14] Gafny S (1999). Spatially and temporally sporadic appearance of macrophytes in the littoral zone of Lake Kinneret, Israel: taking advantage of a window of opportunity. Aquat. Bot..

[15] Lécrivain N (2021). Water-level fluctuation enhances sediment and trace metal mobility in lake littoral. Chemosphere.

[16] Yin D, Peng F, He T, Xu Y, Wang Y (2020). Ecological risks of heavy metals as influenced by water-level fluctuations in a polluted plateau wetland, southwest China. Sci. Total Environ..

[17] Yin D (2020). Production and migration of methylmercury in water-level-fluctuating zone of the Three Gorges Reservoir, China: Dual roles of flooding-tolerant perennial herb. J. Hazard. Mater..

[18] Gownaris NJ, Rountos KJ, Kaufman L, Kolding J, Lwiza KMM, Pikitch EK (2018). Water level fluctuations and the ecosystem functioning of lakes. J. Great Lakes Res..

[19] Liu JF (2019). Water-level fluctuations are key for phytoplankton taxonomic communities and functional groups in Poyang Lake. Ecol. Indic..

[20] Liu Q (2019). Vegetation dynamics under water-level fluctuations: Implications for wetland restoration. J. Hydrol..

[21] Wang P, Zhang Q, Xu Y, Yu F (2016). Effects of water level fluctuation on the growth of submerged macrophyte communities. Flora Morphol. Distrib. Funct. Ecol. Plants..

[22] Yang Z (2020). Discharge and water level fluctuations in response to flow regulation in impounded rivers: an analytical study. J. Hydrol..

[23] Yuan S, Yang Z, Liu X, Wang H (2019). Water level requirements of a *Carex* hygrophyte in Yangtze floodplain lakes. Ecol. Eng..

[24] Zweig CL, Kitchens WM (2009). Multi-state succession in wetlands: a novel use of state and transition models. Ecology.

[25] Bovo-Scomparin VM, Train S (2008). Long-term variability of the phytoplankton community in an isolated floodplain lake of the Ivinhema River State Park Brazil. Hydrobiologia.

[26] Yang Y, Cao Y, Zhang S (2015). Effects of soil moisture regime on rhizomatic germination and young shoot growth of *Carex cinerascens*. J. Ecol. Rural Environ..

[27] Liu L, Liu D, Johnson DM, Yi ZQ, Huang YL (2012). Effects of vertical mixing on phytoplankton blooms in Xiangxi Bay of Three Gorges Reservoir: Implications for management. Water Res..

[28] Souza L (2018). Morphology-based functional groups as the best tool to characterize shallow lake-dwelling phytoplankton on an Amazonian floodplain. Ecol. Indic..

[29] Stević F, Mihaljević M, Špoljarić D (2013). Changes of phytoplankton functional groups in a floodplain lake associated with hydrological perturbations. Hydrobiologia.

[30] Lytle DA, Poff LR (2004). Adaptation to natural flow regimes. Trends Ecol. Evol..

[31] Visser EJW, Bogemann GM, Steeg HMVD, Pierik R, Blom CWPM (2000). Flooding tolerance of *Carex* species in relation to field distribution and aerenchyma formation. New Phytol..

[32] Huber H (2012). Plasticity as a plastic response: How submergence-induced leaf elongation in *rumex palustris* depends on light and nutrient availability in its early life stage. New Phytol..

[33] Fraser LH, Karnezis JP (2005). A comparative assessment of seedling survival and biomass accumulation for fourteen wetland plant species grown under minor water-depth differences. Wetlands..

[34] Deng H (2018). Morphology and physiological characteristics of *Stachys lanata* seedling under water stress. Acta Bot. Boreal.

[35] Tan Z, Zhang Q, Li Y, Xu X, Jiang J (2016). Distribution of typical vegetation communities along elevation in Poyang Lake wetlands. Wetland Sci..

[36] Scholte P (2007). Maximum flood depth characterizes above-Ground biomass in african seasonally shallowly flooded grasslands. J. Trop. Ecol..

[37] Yan B, Dai Q, Liu X, Huang S, Wang Z (2015). Flooding-induced membrane damage, lipid oxidation and activated oxygen generation in corn leaves. Plant Soil..

[38] Shelford VE (1911). Physiological animal geography. J. Morphol..

[39] Luan, Z., Wang, Z., Yan, D., Liu, G., Xu, Y. The ecological response of *Carex lasiocarpa* community in the Riparian Wetlands to the environmental gradient of water depth in Sanjiang Plain, Northeast China. *Sci. World J.* 1–7 (2013). 10.1155/2013/402067 PMC3770044 24065874

[40] Zhang J.T. Quantitative Ecology. Beijing: Science press. 592 (2004).

[41] Zhang M, Chen F, Wu Y, Ma Y, Guan S, Huang Y (2017). Characteristics of the soil seed bank of planted and natural restored draw-down zones in the Three Gorges Reservoir Region. Ecol. Eng..

[42] Ma Y-R, Chen S-H, Chen F-Q, Chen G-H, Xie Z-Q, Liu Y-Y (2018). Effects of flooding on seed viability and nutrient composition in three riparian shrubs and implications for restoration. J. Freshwater Ecol..

[43] Chen F (2019). Impact of regulated water level fluctuations on the sexual reproduction of remnant *Myricaria laxiflora* populations. Glob. Ecol. Conserv..

[44] Webb JA, Wallis EM, Stewardson MJA (2012). systematic review of published evidence linking wetland plants to water regime components. Aquat. Bot..

[45] Deegan BM, White SD, Ganf GG (2007). The influence of water level fluctuations on the growth of four emergent macrophyte species. Aquat. Bot..

[46] Sarneel JM, Janssen RH, Rip WJ, Bender I, Bakker ES (2014). Windows of opportunity for germination of riparian species after restoring water level fluctuations: a field experiment with controlled seed banks. J. Appl. Ecol..

[47] Wei G, Chen Y, Sun X, Chen Y, Luo F, Yu F (2019). Growth responses of eight wetland species to water level fluctuation with different ranges and frequencies. PLoS ONE.

[48] Crosby SC, Ivens-Duran M, Bertness MD, Davey E, Deegan LA, Leslie HM (2015). Flowering and biomass allocation in US Atlantic coast *Spartina alterniflora*. Am. J. Bot..

[49] Mony C, Mercier E, Bonis A, Bouzillé JB (2010). Reproductive strategies may explain plant tolerance to inundation: a mesocosm experiment using six marsh species. Aquat. Bot..

[50] Arias ME, Wittmann F, Parolin P, Murray-Hudson M, Cochrane TA (2016). Interactions between flooding and upland disturbance drives species diversity in large river floodplains. Hydrobiologia.

[51] Gattringer JP, Ludewig K, Harvolk-Schöning S, Donath TW, Otte A (2018). Interaction between depth and duration matters: flooding tolerance of 12 floodplain meadow species. Plant Ecol..

[52] Feng W, Xu L, Wang X, Li H, Jiang J (2016). Response of *Carex cinerascens* populations to groundwater level gradients in the Poyang Lake wetland. Acta Ecol. Sin..

[53] Chen D (2019). A multi-species comparison of selective placement patterns of ramets in invasive alien and native clonal plants to light, soil nutrient and water heterogeneity. Sci. Total Environ..

[54] Liu B (2016). Differential flooding impacts on echinochloa caudata and scirpus planiculmis: implications for weed control in wetlands. Wetlands.

[55] Hao S, Cao H, Wang H, Pan X (2019). The physiological responses of tomato to water stress and re-water in different growth periods. Sci. Hortic. Amsterdam..

[56] Liang F, Huang S, Yu Y, Huang Q, Zhang J, Tan X (2019). Growth and physiological response of *Barringtonia acutangular* to freshwater flooding stress. J. Southw. For. Univ. (Nat. Sci.)..

[57] Plazas M (2019). Comparative analysis of the responses to water stress in eggplant (*Solanum melongena*) cultivars. Plant Physiol. Biochem..

[58] Juan-Ovejero R, Benito E, Barreal ME, Rodeiro J, Briones MJI (2019). Tolerance to fluctuating water regimes drives changes in mesofauna community structure and vertical stratification in peatlands. Pedobiologia.

[59] Wang H, Liu X, Wang H (2016). The Yangtze River floodplain: threats and rehabilitation fishery resources, environment, and conservation in the Mississippi and Yangtze (Changjiang). River Basins.

[60] Casanova MT, Brock MA (2000). How do depth, duration and frequency of flooding influence the establishment of wetland plant communities?. Plant Ecol..

[61] Wang Q, Chen J, Liu H, Yin L, Li W, Liu F (2012). The growth responses of two emergent plants to the water depth. Acta Hydrobiol. Sin..

[62] Zhang Q (2014). An investigation of enhanced recessions in Poyang Lake: comparison of Yangtze River and local catchment impacts. J. Hydrol..

[63] Fang C, Cao W, Mao J, Li H (2018). Relationship between Poyang Lake and Yangtze River and influence of Three Georges Reservoir. J. Hydraul. Eng..

[64] Guo H, Hu Q, Wang Y (2012). Annual variations in climatic and hydrological processes and related flood and drought occurrences in the Poyang Lake Basin. Acta Geogr. Sin..

[65] Hu Z, Fu J (2018). Quantitative study on hydrology relationship between the Yangtze River and Poyang Lake and its changes. J. Hydraul. Eng..

[66] Dai X, Wan R, Yang G, Wang X (2014). Temporal variation of hydrological rhythm in Poyang Lake and the associated water exchange with the Yangtze River. Sci. Geogr. Sin..

[67] Feng L, Hu C, Chen X, Li R, Tian L, Murch B (2011). MODIS observations of the bottom topography and its inter-annual variability of Poyang Lake. Remote Sens. Environ..

[68] Xie F, Wang S (1984). The meadow of poyang lake. Chin. J. Grassland.

[69] Wu Z, Chen X (2004). Flora reipublicae popularis sinicae.

[70] Cao Y, Wang J, Guo Z, Wu H, Luo S (2018). Study on natural distribution and influential factors of *Phalaris Arundinacea* in Poyang Lake National Wetland Park. Yangtze River.

[71] Cao Y, Guo Z, Yang Y, Wang G, Xie Z (2015). The ecological amplitude of acorus calamus young shoots under water level gradient. Pol. J. Ecol..

[72] Liang Q, Wang Z, Lin B, Li C (2019). Effects of flooding stress on seedling physiological indexes and leaf fluorescence characteristics of *Sorbus pohuashanensis*. J. Jilin For. Sci. Technol..

[73] Mommer L, Lenssen JPM, Huber H, Visser EJW, Kroon HD (2006). Ecophysiological determinants of plant performance under flooding: a comparative study of seven plant families. J. Ecol..

[74] Wright SD, Mcconnaughay KDM (2002). Interpreting phenotypic plasticity: the importance of ontogeny. Plant Spec. Biol..

[75] Coleman MCS (1999). Biomass allocation in plants: ontogeny or optimality? A test along three resource gradients. Ecology.

[76] Parolin P (2002). Submergence tolerance vs. escape from submergence: two strategies of seedling establishment in Amazonian floodplains. Environ. Exp. Bot..

[77] Zhang D (2019). Effects of drought and re-flooding on growth and photosynthesis of *Carex schmidtii* Meinsh: Implication for tussock restoration. Ecol. Indic..

[78] Deegan BM, White SD, Ganf GG (2012). Nutrients and water level fluctuations: a study of three aquatic plants. River Res. Appl..

[79] Warwick NWM, Brock MA (2003). Plant reproduction in temporary wetlands: the effects of seasonal timing, depth, and duration of flooding. Aquat Bot..

[80] André M, Blanch S, Grillas P (2001). Effects of submergence on the growth of *Phragmites australis* seedlings. Aquat Bot..

[81] Venterink HO, Wassen MJ, Belgers JDM, Verhoeven JTA (2001). Control of environmental variables on species density in fens and meadows: importance of direct effects and effects through community biomass. J. Ecol..

[82] Hu Z, Ge G, Liu C, Chen F, Li S (2010). Structure of Poyang Lake wetland plants ecosystem and influence of lake water level for the structure. Resour. Environ. Yangtze Basin.

[83] Gause GF (1931). The influence of ecological factors on the size of population. Am. Nat..

[84] Zhang L, Yin J, Jiang Y, Wang H (2012). Relationship between hydrological conditions and vegetation communities in Poyang Lake national nature reserve of China. Adv. Water Sci..

[85] Wu J (2010). Structure analysis of beach vegetation in Poyang Lake in autumn. Jiangxi Sci..

[86] Qi Q (2021). The driving mechanisms for community expansion in a restored *Carex* tussock wetland. Ecol. Ind..

